# Efficacy of Tenofovir-Based Combination Therapy versus Tenofovir Monotherapy in Chronic Hepatitis B Patients Presenting with Suboptimal Responses to Pretreatment: A Meta-Analysis

**DOI:** 10.1155/2016/7214020

**Published:** 2016-01-11

**Authors:** Ling Chen, Xiwei Wang, Qiongfang Zhang, Jiaojiao Gong, Shasha Shen, Wenwei Yin, Huaidong Hu

**Affiliations:** Department of Infectious Diseases, Institute for Viral Hepatitis, Key Laboratory of Molecular Biology for Infectious Diseases, Ministry of Education, Second Affiliated Hospital of Chongqing Medical University, Chongqing 400016, China

## Abstract

*Background/Aims*. It remains unclear whether tenofovir disoproxil fumarate- (TDF-) based combination therapy produces better outcomes than TDF monotherapy in chronic hepatitis B (CHB) patients. The aim of this study was to compare the efficacy of the two regimens by performing a meta-analysis. *Methods*. A comprehensive literature search was performed on the comparison of TDF-based combination therapy and monotherapy for CHB patients in the PubMed, Embase, Web of Science, and the Cochrane Libraries. Both dichotomous and continuous variables were extracted and pooled outcomes were expressed as risk ratio (RR) or standard mean difference (SMD). *Results*. Nine eligible studies (1089 subjects in total) were included in our analysis. The proportion of patients with undetectable HBV DNA at 24, 48, and 96 weeks were similar between the two comparable groups (62.5% versus 70.9%, *P* = 0.086; 78.1% versus 83.7%, *P* = 0.118; 86.4% versus 87.9%, *P* = 0.626, resp.). HBV DNA reduction, rates of ALT normalization, hepatitis B e antigen (HBeAg) loss, and HBeAg seroconversion were also similar between the two groups. *Conclusions*. On the current data, TDF-based combination therapy seemed to be no better than those achieved by monotherapy. Further studies are needed to verify this comparison.

## 1. Introduction

Hepatitis B virus (HBV) infection is a worldwide healthcare problem and HBV virions cannot be cleared completely because the covalently closed circular DNA persists in the nuclei of infected hepatocytes. Hence, the main purpose of antiviral therapy is sustained viral suppression. Guidelines recommend sustained viral suppression as the most effective way to reduce complications and improve quality of life [1]. Antiviral therapy with nucleos(t)ide analogues (NAs) is now the frontline treatment for chronic hepatitis B (CHB) patients because of its demonstrably suppressive effects on viruses. However, NAs must be administered for extended periods due to the high frequency of virological relapse following the discontinuation of treatment, but long-term use of NAs can result in drug resistance and toxicity [2, 3]. Therefore, optimal antiviral treatments with a high genetic barrier to resistance, good safety, and durable efficacy should be deployed [4].

Currently, TDF is a potent inhibitor of HBV replication with a high genetic barrier to resistance among both treatment-naive and NAs-experienced CHB patients [5, 6]. Even though other NAs are effective in lowering HBV DNA levels and improving prognosis after long-term treatment, treatment resistance has been reported in 70% of patients after four years of lamivudine (LAM) treatment, 29% of HBV e antigen- (HBeAg-) negative patients after five years of Adefovir dipivoxil (ADV) treatment, 25% of HBeAg-negative patients after two years of telbivudine (LdT) treatment, and 1.2% of patients after five years of entecavir (ETV) treatment [7–10]. Conversely, there has been no associated resistance observed after three years of TDF treatment [6]. In addition, treatment with TDF for up to six years leads to a significant decrease in hepatitis B surface antigen (HBsAg) in the HBeAg-positive population with HBV/HIV coinfection: cumulative rates of HBsAg seroclearance were 8% in these cases; such high rates of HBsAg seroclearance are not achieved by other NAs [11].

TDF monotherapy is effective for patients with viral breakthrough or having suboptimal responses to previous NA treatment, but TDF-based combination therapy should be considered in patients with drug resistance because of a further decrease in HBV DNA following the addition of another NA [12]. However, it remains a subject of controversy whether TDF-based combination therapy induces better outcomes than TDF monotherapy in NA-experienced patients with previous treatment failures. No relevant meta-analyses have directly compared the two treatment strategies. Thus, our meta-analysis aimed to do so, comparing the relative efficacy of TDF-based combination therapy and TDF monotherapy in CHB patients with previous treatment failures.

## 2. Methods

### 2.1. Literature Search

Relevant studies regarding the comparison of TDF-based combination therapy and TDF monotherapy for CHB patients were identified by searching the PubMed, Embase, Web of Science, and the Cochrane Libraries, using the medical subject headings “tenofovir”, “chronic hepatitis B”, “monotherapy”, “combination therapy”, “nucleoside analog”, “nucleotide analog”, and their abbreviations. Multiple synonyms were also used. The search was restricted to “human” and “English”. The reference lists of all the retrieved documents were manually searched for potentially relevant reports missed by the intelligent retrieval systems mentioned above. The search was carried out in March, 2015, and the entire selection process was completed independently by two investigators (LC and XWW). Inconsistent search results were resolved with the assistance of an arbiter (HDH) where necessary.

### 2.2. Selection Criteria

Inclusion criteria for the meta-analysis were as follows: (a) randomized controlled trials (RCTs), retrospective and prospective cohort study designs; (b) patients with CHB (defined as a positive serum HBsAg test for at least 6 months) having previously received any NA other than TDF and presenting with a suboptimal response to the prior NA treatment; (c) studies comparing TDF-based combination therapy and TDF monotherapy for previously treated HBV with a course of therapy equal to or more than 48 weeks; and (d) studies providing information that included, at minimum, virological response (HBV DNA levels), serological response (HBeAg and HBsAg loss or seroconversion), or biochemical response (ALT normalization). Studies were excluded if they featured (a) noncomparative data or observational methodologies, (b) no available outcome measures and a therapy course of less than 48 weeks, (c) coinfection with hepatitis A, hepatitis C, hepatitis D, or hepatitis E viruses or human immune-deficiency virus (HIV), and (d) definitive diagnosis with HCC or a history of renal failure and organ transplantation.

### 2.3. Outcome Measures

The rates of virological response, biochemical response, and serological response were used as primary efficacy measures. “Virological response” included virological suppression defined as achievement of undetectable HBV DNA levels to below the detection level and HBV DNA levels that changed over time. “Biochemical response” included ALT normalization, defined as the proportion of subjects with normal ALT levels after treatment, where patients had had abnormal ALT levels at baseline. “Serological response” included rates of HBeAg loss, HBeAg seroconversion, HBsAg loss, and HBsAg seroconversion. The adverse effects (AEs) caused by study drugs also received special attention.

### 2.4. Data Extraction

All data were independently extracted from the included studies by two investigators (LC and XWW) and, where possible, calculated and checked twice. Any dispute between investigators was resolved by discussion or arbitration (by HDH) when necessary. If useful data were presented indirectly by figures or graphs, they were translated into correlative patterns by using Get-Data software or relevant formulae when there was no response from authors. If mean values or standard deviation (SD) for analysis was unavailable, they were calculated from medians and ranges using relevant formulae [13]. The following information was extracted: year of publication, study design, race of participants, number of patients per study group, patient clinical characteristics at baseline, treatment regimen and course received, and interesting endpoints.

### 2.5. Study Quality

The quality of all included RCTs was assessed using the revised Jadad quality scale, which graded the quality of a study from 0 (lowest) to 7 (highest) by examining randomization, blinding, allocation concealment, and drop-out. For cohort designs, the quality was assessed using the Newcastle-Ottawa Scale (NOS) based on several standards including selection of cohorts, comparability of cohorts, and assessment of the outcomes.

### 2.6. Statistical Analysis

Data analysis was carried out with software Stata version 12.0 (Stata Corporation, College Station, TX, USA) and was based on an intent-to-treat principle. Both dichotomous and continuous variables were extracted in this analysis. Outcomes were expressed as RR, or SMD with 95% confidence intervals (CI). The overall effects were measured using a *Z*-score with a significance set at *P* < 0.05. Statistical heterogeneity was evaluated by using chi-square and *I*-square (*I* ^2^) tests with a significance set at *P* < 0.1. *P* < 0.1 and *I* ^2^ > 50% were considered to be significant heterogeneity. The random-effect method was used to combine results if confirmed significant heterogeneity was observed; otherwise, the fixed-effect method was used. To assess sources of potential bias, sensitivity analyses were performed where required. The publication bias of selected articles was assessed by funnel plots and any potential bias was judged by Begg's and Egger's tests.

## 3. Results

### 3.1. Search Results

**Figure 1:**
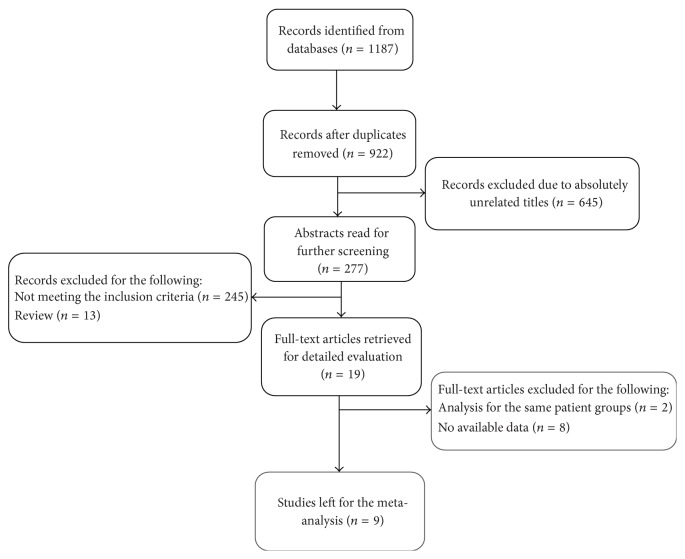
Flow diagram of literature selection process.

The search strategy resulted in the identification of 1187 records in total. 265 records were duplicate documents retrieved from two or more databases and thus removed. The remaining studies received further screening by scanning the title or abstract, which resulted in the exclusion of a further 903 studies. As a result, 19 full-text articles were subjected to detailed evaluation, of which two were excluded because they analyzed the same patient groups, and a further eight were excluded due to lack of available data. Eventually, nine eligible articles relating to a total of 1089 subjects (592 in combination therapy groups and 497 in monotherapy groups) were chosen for this meta-analysis (Figure 1).

Of the nine eligible studies, five were RCTs [14–18] and four were cohorts [19–22]. All of the five RCTs receiving a Jadad score of at least 5 were considered of relatively high quality and all of the four cohort studies received NOS score of at least 5 (Supplementary Table 1 in Supplementary Material available online at http://dx.doi.org/10.1155/2016/7214020). The detailed characteristics of the included studies are summarized in Table 1. For those endpoints with more than five included articles, we performed analysis of publication bias. Publication bias was not found in any outcome measure (Supplementary Figure 1).

### 3.2. Virological Responses

**Figure 2:**
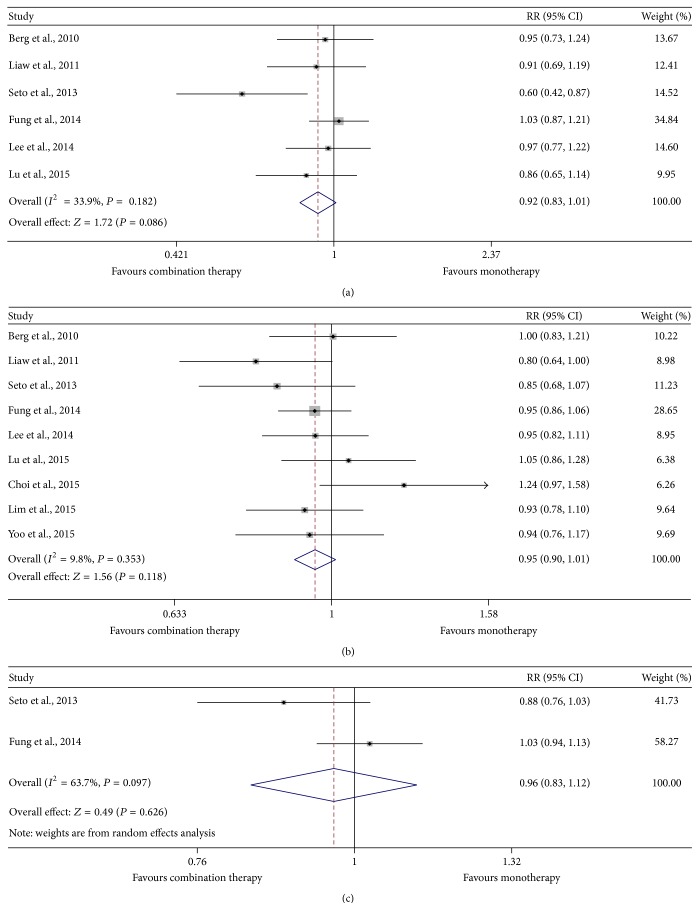
Forest map of summary estimates for comparison of virological suppression between TDF-based combination therapy and monotherapy groups. (a) 24 weeks; (b) 48 weeks; (c) 96 weeks.

Rates of undetectable HBV DNA were similar between TDF monotherapy and combination therapy at 24, 48, and 96 weeks (62.5% versus 70.9%, *P* = 0.086; 78.1% versus 83.7%, *P* = 0.118; 86.4% versus 87.9%, *P* = 0.626, resp.) (Figure 2). Six studies with a total of 583 patients reported a change of serum HBV DNA levels at 48 weeks from baseline and no superior efficacy was demonstrated in TDF-based combination therapy when compared to monotherapy (*P* = 0.459) (Supplementary Figure 2).

### 3.3. Serological Response

**Figure 3:**
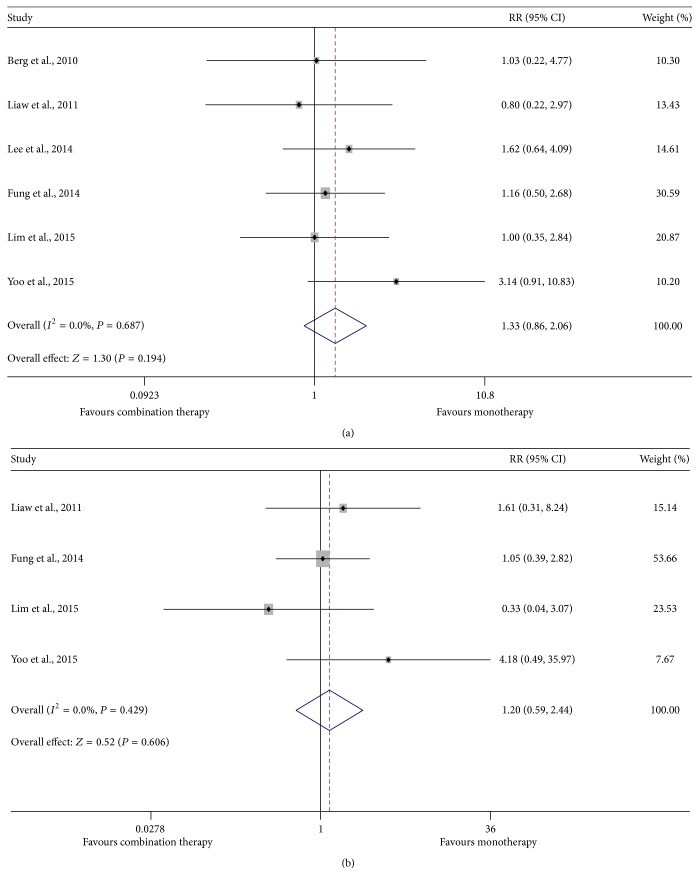
Forest map of summary estimates for comparison of the changes in HBeAg between TDF-based combination therapy and monotherapy groups. (a) HBeAg loss; (b) HBeAg seroconversion.

Among HBeAg-positive patients, data for HBeAg loss and seroconversion analysis were extracted and a fixed-effect model showed that a similar proportion in each treatment group experienced HBeAg loss (16.4% versus 14.1%, *P* = 0.194) and seroconversion to anti-HBe (9.2% versus 7.7%, *P* = 0.606) during the study period (Figure 3). Three patients (two in TDF monotherapy groups and one in TDF-based combination therapy groups) were reported to have achieved HBsAg seroclearance but only one of these obtained seroconversion to anti-HBs.

### 3.4. Biochemical Response

**Figure 4:**
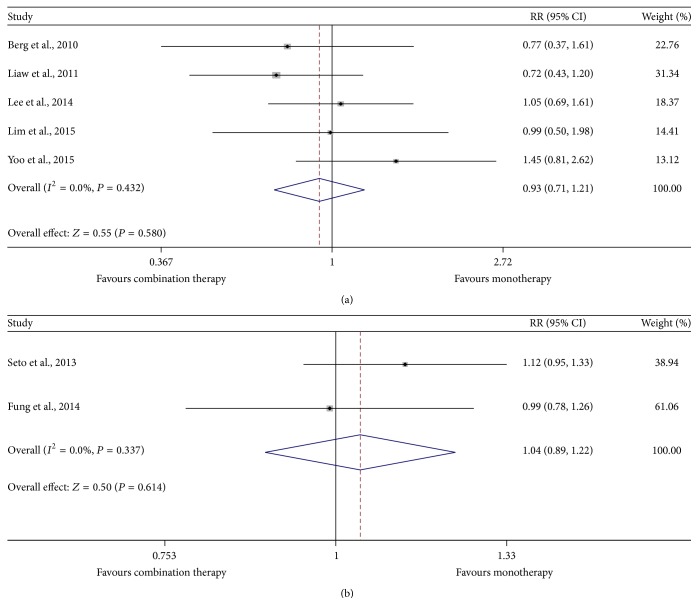
Forest map of summary estimates for comparison of ALT normalization between TDF-based combination therapy and monotherapy groups. (a) 48-week ALT normalization; (b) 96-week ALT normalization.

Among those patients with abnormal ALT levels at baseline, data about ALT normalization were exacted and a fixed-effect model showed that a similar proportion of patients in the two groups experienced ALT normalization at each time point (44.4% versus 50.4%, *P* = 0.580 for 48 weeks; 74.6% versus 68.4%, *P* = 0.614 for 96 weeks) (Figure 4).

### 3.5. Safety

Berg et al. [14] reported that, in the combination group, one patient suffered from severe study drug-related AE with an increase of ALT from 80 U/L at baseline to 432 U/L at week 8. Fung et al. [16] reported that three patients (two in monotherapy group and one in combination group) experienced study drug discontinuation for AEs, of which one was judged to be caused by the study drug. Liaw et al. [15] reported that six patients dropped out of the study because of AEs but none of these AEs was considered to be related to study drug. Out of all included patients, six patients developed HCC and six patients experienced bone fracture, but none of these cases was considered to be study treatment related.

## 4. Discussion

Even though TDF exhibited effective and safe outcomes in patients with previously multiple NA treatment failures [23], HBV DNA decline was slower in NA-experienced patients than in treatment-naive patients after TDF therapy; optimal TDF-based combination treatment should thus be considered in NA-experienced patients [24]. TDF-emtricitabine (FTC) combination therapy resulted in undetectable HBV DNA levels without any renal toxicity for those with detectable HBV DNA on ADV [25]. Rescue therapy with TDF-ETV combination was efficient and safe in patients with multidrug-resistant (MDR) HBV strains regardless of the antiviral drug resistance profiles [26]. LdT therapy with TDF intensification in HBeAg-positive CHB patients at week 24 appeared effective and well tolerated [27]. Both TDF monotherapy and combination therapy can effectively inhibit the virus and show good levels of tolerability in NA-experienced patients, but which one can produce better outcomes is still uncertain. Therefore, we performed a meta-analysis including studies that involved the comparison between TDF-based combination therapy and monotherapy, to investigate the uncertainty.

Despite the fact that TDF-based combination therapy is reported to provide more effective HBV suppression than therapy with each drug alone in vitro and in a robust mouse model [28], the results of our meta-analysis suggest that viral suppression occurring in monotherapy groups seemed to be similar to that in combination therapy groups (62.5% versus 70.9%, *P* = 0.086 at 24 weeks; 78.1% versus 83.7%, *P* = 0.118 at 48 weeks; 86.4% versus 87.9%, *P* = 0.626 at 96 weeks). Rates of undetectable HBV DNA were all higher in TDF combination therapy groups at the three time points but none of the three time points achieved statistical significance. According to these data, we can find that the difference of the rates of undetectable HBV DNA between the two regimens was gradually narrowing with the follow-up time extending. The reason for this tendency may be primarily due to the gradual emergence of resistance induced by study drug that TDF combined with in combination therapy groups.

Even though significance was not achieved, our meta-analysis demonstrated that combination therapy tended to lead to less HBeAg loss, and this was contrary to the result of undetectable HBV DNA, for which the reason may be that combination groups contained more HBeAg-positive patients than those that monotherapy groups included at baseline (as shown in Table 1), thereby resulting in lower HBeAg loss rates in combination groups in the case that the absolute numbers of HBeAg loss during study were not much different between the two comparative groups. Only three patients were reported to have HBsAg loss, a number far below that reported by Zoutendijk et al. in the HBeAg-positive population where HBV/HIV coinfection received treatment of TDF for up to 6 years [11]. In our study, of the included patients, all were HBV monoinfected and only a portion were HBeAg-positive. Additionally, the longest duration of TDF treatment reported in the studies included in our analysis was 3 years, which is far shorter than 6 years; these may be the reasons for the low HBsAg loss rate in our meta-analysis.

Drug safety and resistance should be seriously considered when switching to new therapeutics in patients who have experienced pretreatment failures. After up to 144 weeks of exposure to TDF monotherapy, no NA-experienced patient developed HBV pol/RT mutations associated with TDF resistance, and a favorable safety profile was maintained in CHB patients [6, 29]. Although renal toxicity is associated with TDF in HIV-infected patients, renal dysfunction was rarely reported in CHB patients [30]. TDF-based combination therapy also had a good safety profile in CHB patients after a median follow-up of >76 weeks [31]. In our meta-analysis, no included studies reported resistance mutations related to TDF management, and virological breakthrough rarely happened in either treatment group. Most study drug-related AEs were gentle and rarely resulted in discontinuation of treatment (only two instances were reported). Berg et al. [14] reported no statistically significant difference in any AE parameter including study drug-related AEs between TDF monotherapy and combination therapy at 48 weeks, with Fung et al. [16] and Yoo et al. [18] reporting similar results at 96 weeks. These results revealed that TDF therapy was well tolerated and safe for NA-experienced patients, while TDF-based combination therapy seemed not to increase the risk of AEs when compared with TDF monotherapy. TDF-based combination therapy resulted in lower adherence to antiviral treatment and higher costs for CHB patients [32], so monotherapy seems to be an optimal choice when patients need to switch to TDF treatment. However, our analysis only covered the period up to 96 weeks; for those patients who need to receive antiviral treatments for a longer time, comparisons of efficacy and safety between TDF-based combination and monotherapy should be conducted using larger and lengthier clinical trials.

Confirmed heterogeneity was found in two endpoints: 48-week HBV DNA reduction (*P* = 0.025, *I* ^2^ = 61.1%) and 96-week virological suppression (*P* = 0.097, *I* ^2^ = 63.7%). Sensitivity analysis was performed to find the source of heterogeneity in the endpoint of HBV DNA reduction. When the study reported by Lee et al. was excluded, the pooled value changed apparently, and the heterogeneity disappeared (*P* = 0.329, *I* ^2^ = 13.4%). We observed that patients included by Lee at al. [22] had clearly lower mean HBV DNA viral load at baseline than those in the five other studies, which may be the reason for the heterogeneity. Only two included articles had a virological suppression endpoint of 96 weeks, of which one, reported by Seto et al. [19], lacked adequate baseline data, making it difficult to locate the source of heterogeneity.

The limitations of this meta-analysis include the fact that the number of trials meeting the inclusion criteria was small and some studies were not RCTs, with three being retrospective designs. Some studies had small sample sizes and one study had no sufficient baseline information, as mentioned above. In addition, the limited number of studies used in analysis of some endpoints may have weakened the statistical power of the meta-analysis and further undermined the ability to evaluate treatment effects. Some endpoints were observed to suffer from heterogeneity, which may have affected the accuracy of these pooled values. Finally, there was no common detection limit for HBV DNA (three used 69 IU/mL, four used 60 IU/mL, and two used 20 IU/mL), but this situation was difficult to avoid.

In conclusion, based on the available data, our results indicate that TDF-based combination therapy did not show any significant advantage in those efficacy indicators nor did it result in any compromised safety when compared to TDF monotherapy. Further studies are needed to verify this comparison.

## Supplementary Material

Table S1 shows the evaluation of included articles' quality. Five RCTs receiving a Jadad score of at least 5 were considered of relatively high quality and the other four cohort studies received NOS score of at least 5.
Figure S1 shows the publication bias of included studies. For those endpoints with more than five included articles, we performed analysis of publication bias. Publication bias was not found in any outcome measure.
Figure S2 shows the comparison of serum HBV DNA reduction between TDF-based combination therapy and monotherapy groups. Six studies reported a change of serum HBV DNA levels at 48 weeks from baseline and no superior efficacy was demonstrated in TDF-based combination therapy when compared to monotherapy.

## Figures and Tables

**Table 1:** Characteristics of studies included in the meta-analysis.

Study	Centers	Design	*N*	Age (year)	Sex (M/F)	Positive HBeAg (*N*)	HBV DNA (log⁡10 copies/mL)	Regimen	Therapy duration (week)	Treatment experience	Resistant mutations
Berg et al. [14]	Multi	RCT	M: 53	40 ± 11.4	38/15	38	6.06 ± 1.43	TDF 300 mg/d	48	LAM, ADV	rtM204V/I, rtL180M, rtV173L, rtN236T, rtA181V/T
			C: 52	39 ± 10.4	42/10	39	5.87 ± 1.78	TDF 300 mg/d; FTC 200 mg/d			

Liaw et al. [15]	Multi	RCT	M: 45	52 ± 2.3	37/8	19	5.7 ± 0.43	TDF 300 mg/d	48	LAM, ADV	rtM204V/I, rtL180M
			C: 45	50 ± 4	40/5	23	6.28 ± 0.7	TDF 300 mg/d; FTC 200 mg/d			

Seto et al. [19]	Single	Cohort	M: 71	NA	NA	NA	NA	TDF 300 mg/d	144	LAM, LdT, ETV, ADV	rtA181V/T, rtN236T, rtA194T
			C: 54	NA	NA	NA	NA	TDF 300 mg/d; LAM 100 mg/d			

Lee et al. [22]	Single	Cohort	M: 33	54 ± 10.8	22/11	16	2.83 ± 1.62	TDF 300 mg/d	48	LAM, ADV	rtM204V/I, rtL180M
			C: 120	54 ± 8.3	84/36	80	2.77 ± 1.09	TDF 300 mg/d; LAM 100 mg/d			

Fung et al. [16]	Multi	RCT	M: 141	47.1 ± 13.6	104/37	65	5.64 ± 1.83	TDF 300 mg/d	96	LAM	rtM204V/I, rtL180M
			C: 139	46.3 ± 13.6	107/32	68	5.77 ± 1.97	TDF 300 mg/d; FTC 200 mg/d			

Lu et al. [20]	Multi	Cohort	M: 25	40 ± 14	16/9	22	3.10 ± 0.95	TDF 300 mg/d	48	ETV	NA
			C: 43	40 ± 10.8	27/16	41	3.57 ± 0.90	TDF 300 mg/d; ETV 1 mg/d			

Choi et al. [21]	Single	Cohort	M: 34	48 ± 8	23/11	26	4.76 ± 1.7	TDF 300 mg/d	48	LAM, LdT, ETV, ADV	rtM204V/I, rtL180M, rt181A, rt236T, rtT184, rtI169, rtS202
			C: 42	50 ± 13	33/9	36	4.54 ± 1.76	TDF 300 mg/d; NAs			

Lim et al. [17]	Single	RCT	M: 45	51 ± 9	32/13	40	4.09 ± 0.6	TDF 300 mg/d	48	LAM, LdT, ETV, ADV	rtM204V/I, rtL180M, rt236T, rtT184, rtI169T, rtS202G, rtM250L/V
			C: 45	52 ± 10	36/9	40	3.74 ± 0.46	TDF 300 mg/d; ETV 1 mg/d			

Yoo et al. [18]	Single	RCT	M: 50	49 ± 10	42/8	44	3.27 ± 1.8	TDF 300 mg/d	96	LAM, LdT, ETV, ADV	rtM204V/I, rtL180M, rtA181T/V, rtN236T, rtT184, rtS202G, rtM250 L/V, rtM204V/I
			C: 52	50 ± 11	46/6	46	3.50 ± 1.69	TDF 300 mg/d; ETV 1 mg/d			

M: monotherapy group; C: combination therapy group; TDF: tenofovir disoproxil fumarate; FTC: emtricitabine; LAM: lamivudine; ETV: entecavir; ADV: adefovir dipivoxil; LdT: telbivudine; RCT: randomized controlled trial; and NA: not available.
