# Supplementary material for: Efficacy of Tenofovir-Based Combination Therapy versus Tenofovir Monotherapy in Chronic Hepatitis B Patients Presenting with Suboptimal Responses to Pretreatment: A Meta-Analysis
Source: Gastroenterol Res Pract. 2016 Jan 11;2016:7214020. doi: 10.1155/2016/7214020 (PMC4737451; doi:10.1155/2016/7214020)

Supplementary Table 1. The quality of studies assessed by the revised Jadad or NOS.

Study	Randomization	Allocation concealment	Blinding	Dropout	Jadad score
Berg et al.[14]	2	2	2	1	7
Liaw et al.[15]	2	1	1	1	5
Fung et al.[16]	2	1	2	1	6
Lim et al.[17]	2	2	0	1	5
Yoo et al.[18]	2	2	0	1	5
**Study**	**Selection**	**Comparability**	**Outcomes**		**NOS score**
Seto et al.[19]	3	1	3		7
Lee et al.[22]	2	1	2		5
Lu et al.[20]	3	1	2		6
Choi et al.[21]	3	1	2		6

Supplementary Figure 1. Funnel plots for some outcomes. A) 24-week virological suppression; B) 48-week virological suppression; C) HBV DNA reduction; D) HBeAg loss.

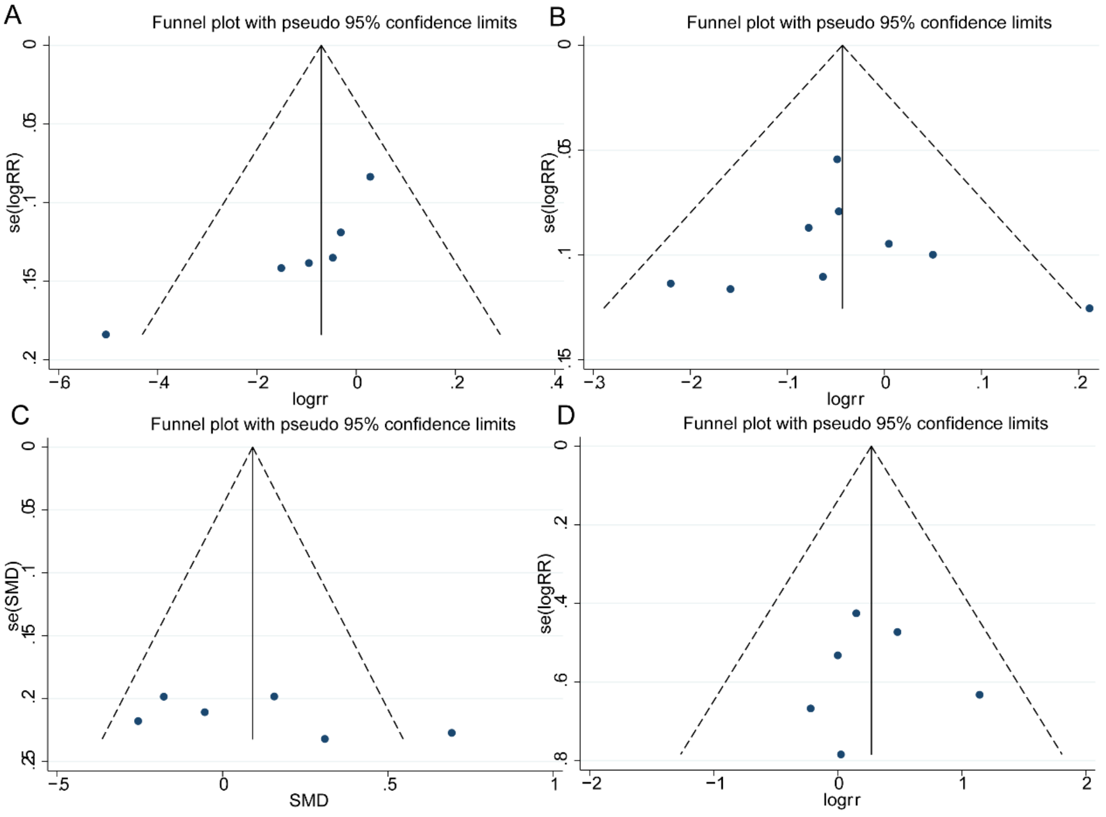

Supplementary Figure 2. Forest map of summary estimate for comparison of serum HBV DNA reduction between TDF-based combination therapy and monotherapy groups.

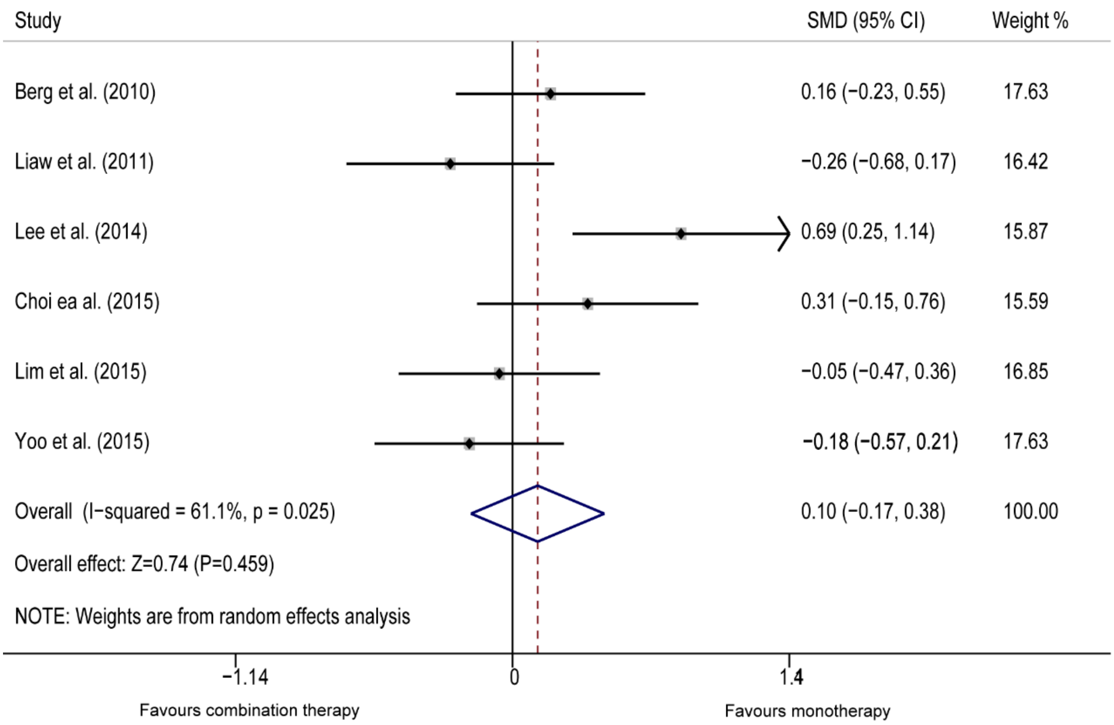

Supplement: Supplementary file 1 — Table S1 shows the evaluation of included articles' quality. Five RCTs receiving a Jadad score of at least 5 were considered of relatively high quality and the other four cohort studies received NOS score of at least 5. Figure S1 shows the publication bias of included studies. For those endpoints with more than five included articles, we performed analysis of publication bias. Publication bias was not found in any outcome measure. Figure S2 shows the comparison of serum HBV DNA reduction between TDF-based combination therapy and monotherapy groups. Six studies reported a change of serum HBV DNA levels at 48 weeks from baseline and no superior efficacy was demonstrated in TDF-based combination therapy when compared to monotherapy. [file 7214020.f1.pdf]
